# Spatial–numerical associations in the presence of an avatar

**DOI:** 10.1007/s00426-020-01424-y

**Published:** 2020-10-07

**Authors:** C. Böffel, C. Herbst, O. Lindemann, J. Müsseler

**Affiliations:** 1 RWTH Aachen University, Aachen, Germany; 2 Erasmus University Rotterdam, Rotterdam, The Netherlands

## Abstract

When we interact with other people or avatars, they often provide an alternative spatial frame of reference compared to our own. Previous studies introduced avatars into stimulus–response compatibility tasks and demonstrated compatibility effects as if the participant was viewing the task from the avatar’s point of view. However, the origin of this effect of perspective taking remained unclear. To distinguish changes in stimulus coding from changes in response coding, caused by the avatar, two experiments were conducted that combined a SNARC task and a spontaneous visual perspective taking task to specify the role of response coding. We observed compatibility effects that were based on the avatar’s perspective rather than the participants’ own. Because number magnitude was independent of the avatar’s perspective, the observed changes in compatibility caused by different perspectives indicate changes in response coding. These changes in response coding are only significant when they are accompanied by visual action effects.

## Introduction

In many everyday situations, we experience conflicting visual perspectives. Imagine, for example, sitting at the dinner table and asking a person sitting opposite you to pass you the hot sauce. Is it to their right or to their left? To solve this problem, we have to adopt the other person’s point of view and imagine the sauce from their perspective. Figuratively speaking, we intend to see the table through their eyes. This change in perspective can be associated with cognitive costs such as increased reaction times or error rates and is often described as visual perspective taking (e.g., Flavell, 1978) or mental self-rotation (e.g., Surtees, Apperly, & Samson, 2013). How demanding perspective taking is depends on the situation. But we generally observe increased costs, when the angular disparity between oneself and the target is sufficiently large (e.g., Janczyk, 2013; Müsseler, Ruhland, & Böffel, 2019). For successful perspective taking, we have to overcome our general tendency to code spatial information from our own perspective (Gardner & Potts, 2011; Taylor, Flynn, Edmonds, & Gardner, 2016) and adopt a new one instead. In a series of experiments, Kessler and Thomson (2010) presented evidence that this process is impacted by embodiment effects by showing that a person’s posture influences perspective taking. When the participants’ body orientation was congruent with the direction of the perspective change, their responses were faster compared to a neutral condition. When it was incongruent, responses were the slowest. In a later follow-up study, Kessler and Wang (2012) examined interpersonal differences regarding this embodied nature of perspective taking and identified two groups of people: “systemisers” who show only weak embodiment effects and “embodiers” who demonstrated high levels of embodiment.

Various paradigms are used to quantify how different perspectives influence our perceptions and actions. The most common tasks ask participants to perform spatial judgments from another person’s point of view, for example by asking them to judge whether an object is on a person’s left or right by pressing the corresponding key on a keyboard. However, these tasks generally do not include trials in which the participants have to perform spatially non-corresponding responses from another viewpoint, i.e., indicating the side where the object is not. Since spatial correspondence generally leads to stimulus–response compatibility, the influence of spatial stimulus–response compatibility effects for perspective taking should be discussed (May & Wendt, 2013).

## Stimulus–response compatibility

Some mappings of stimuli to responses consistently yield an improved performance compared to others, a phenomenon known as stimulus–response compatibility (SRC). For example, a left key press is usually compatible with a stimulus presented to the left ear, while a right response would be incompatible and slower. But stimulus–response compatibility is not limited to the spatial dimension and does occur in a wide variety of situations (for overviews see Kornblum, Hasbroucq, & Osman, 1990; Proctor & Vu, 2006).

Kornblum et al. (1990) provide a model to explain stimulus–response compatibility: the dimensional overlap model. It proposes that SRC is a result of an overlap between stimulus and response dimensions that causes the automatic activation of a corresponding response. This automatic activation constitutes the direct route to response activation, while the retrieval of the mapping rule constitutes the indirect route. A conflict of both routes impairs performance. It is important to note that a dimensional overlap has to be present for the automatic route to take effect. Otherwise, if there is no dimensional overlap the direct route is incapacitated.

The dimensional overlap model is straightforward when it comes to basic stimulus–response compatibility tasks where the location often provides the dimensional overlap. However, a dimensional overlap can also occur on a more abstract level, for example, when a right response and an upper stimulus position both overlap in the abstract dimension polarity, or salience (Weeks & Proctor, 1990). Some examples of stimulus–response compatibility are based on an abstract dimensional overlap, for example associations. Such associations are, among others, of space and size (Wühr & Seegelke, 2018), space and valence (Casasanto & Chrysikou, 2011; Kong, 2013), and famously, space and numerical magnitude (Dehaene, Bossini, & Giraux, 1993).

## Spatial–numerical associations

A well-known example of a stimulus–response compatibility effect that is based on culturally acquired associations is the so-called SNARC effect (spatial numerical associations of response codes, Dehaene et al., 1993; Fias & Fischer, 2005). Dehaene et al. (1993) demonstrated in a series of experiments that smaller numbers were compatible with left side responses, while large numbers were compatible with right side responses. This effect also occurred after the participants crossed their hands (Dehaene, Bossini, & Giraux, 1993, Exp. 6), although this result has been called into question by a more recent replication study (Wood, Nuerk, & Willmes, 2006).

To explain the spatial–numerical stimulus–response compatibility effect, several authors have used the metaphor of a “mental number line” and argued that numbers are represented spatially along a mental continuum, where—in participants from western cultures—larger numbers are located on the right side of space and smaller numbers on the left.

Interestingly, it has been shown that the spatial coding of numbers is rather flexible (Fischer, 2006; Lindemann, Abolafia, Pratt, & Bekkering, 2008). For instance, spatial–numerical associations have been found between numbers and responses along the sagittal axis, that is, for responses that were characterized by either a button press close or far from the body (Ito & Hatta, 2004; Shaki & Fischer, 2012). For instance, Ito and Hatta (2004) observed a sagittal SNARC effect in Japanese participants and showed that large numbers were associated with being far from the body and small numbers with close responses. Although some authors therefore concluded that the numbers are spontaneously mapped onto all dimensions in space, there is only little evidence for the existence of real egocentric vertical SNARC effects from number classification tasks with manual responses (Wiemers, Bekkering, & Lindemann, 2014). Several studies have been reported that failed to find vertical number–space compatibility effects (Holmes & Lourenco, 2012; Wiemers, Bekkering, & Lindemann, 2017). For instance, Hartmann, Gashaj, Stahnke and Mast (2014) examined vertical SNARC effects and found a significant up-to-down mapping only in one of their four experiments. Wiemers et al. (2017) recently reviewed the literature and showed that most evidence incorrectly interpreted as support for vertical SNARC effects are based on studies using responses arranged on the sagittal axis or on studies that confound vertical positions with horizontal anatomical hand mappings. However, in a recent study, Aleotti et al. (2020) were able to show SNARC effects for all three dimensions, including a vertical effect.

It should be emphasized that cognitive associations with sagittal and vertical dimensions must be distinguished conceptually. Theories about the origin of vertical number–space mappings often propose a geo- or allocentric spatial reference frame that might be based on observations in nature, e.g., that a quantity of items stack up to a certain amount (for a discussion about possible origins of different numerical–spatial associations, see Winter, Matlock, Shaki, & Fischer, 2015). In contrast, horizontal and sagittal spatial–numerical associations, for which the literature is providing a large body of evidence, are based on egocentric spatial codes that are relative to one’s own body (such left, right, close or far). One proposed reason for this is the mental representations of time in which the self is in the center, representing the present. The future lies on the right, the past on the left (cf. Bonato, Zorzi, & Umiltà, 2012) and events further away are associated with greater physical distance on a sagittal axis (Marghetis & Youngstrom, 2014).

The present study aims to examine the effects of automatic perspective taking on the SNARC effect to gain insights about changes in response coding caused by an avatar and its perspective. For this, spatial–numerical associations are useful, because they allow us to use numbers as stimuli that have a spatial component by causing associations on a left-to-right dimension, without the need to occupy different positions on that axes in physical space. In contrast, in the typical Simon tasks, stimuli gain their spatial features because they occupy space on either side of the body. Furthermore, the magnitude of the number is independent of perspective, while the positions of a stimulus in space can change based on perspective. That is, within the number range of 1–9, the number 1 is always the smallest and 9 the largest number, regardless of the perspective used to view it. Also, the cognitive preference to associate small numbers with the left and large numbers with right egocentric space is not affected by the perspective. This feature allows us to investigate other perspective-based changes, such as changes in response codes, while the magnitude of the number remains unaltered by perspective changes. We hypothesized that if a person is presented with an avatar, the avatar serves as a reference frame of the number–space mapping, by allowing the same response to be either coded as left or right, depending on the avatar’s perspective. Effects of spatial–numerical association should consequently depend on the avatar’s orientation. In experiments with real confederates, it was demonstrated that participants can share a mental number line (Hartmann, Fischer, & Mast, 2019) and that the presence of another person and their action are represented while sharing a SNARC task (Atmaca, Sebanz, Prinz, & Knoblich, 2008). However, it is unclear if similar effects can be induced by an avatar that additionally provides a different perspective. Since an avatar is generally a representation of the user who often controls the avatar, the person–avatar interaction can be regarded as less social and therefore creates less demand to infer the mental states of the avatar compared to the interaction of two persons. To investigate whether control over the avatar is a requisite for potential changes in response codes, the avatar will be controlled by the participants in Experiment one, but static in Experiment 2.

## Stimulus–response compatibility as an indicator of cognitive representation

The idea of using SRC effects to examine other cognitive phenomena is not new (e.g., Hommel, 2011) and has already been used in the context of perspective taking to quantify the influence of a conflicting reference frame, either of a person (Freundlieb, Kovács, & Sebanz, 2016), an avatar (Böffel & Müsseler, 2018, 2019a, b, 2020a, b;  Müsseler et al., 2019) or even simple manikins (Baess, Weber, & Bermeitinger, 2018). Because SRC has been shown to be dependent on context and intention (Hommel, 1993; Müsseler, Aschersleben, Arning, & Proctor, 2012), it seems to emerge based on the mental representation of an event, rather than on its mere physical reality. Changes in stimulus–response compatibility can therefore be used to quantify changes in the mental representation of certain aspects of an event, for example the location of a stimulus. Böffel and Müsseler (2019b) give an example for this, as stimuli that were presented at the top or bottom of the screen produced a compatibility effect as if they were regarded as left or right, depending of the perspective of an avatar. The interpretation was that the vertically presented stimuli gained horizontal features, for example changing the mental representation of a bottom stimulus to a left one, if presented on the left of an avatar. We also can try to estimate how large this change is. If the observed compatibility effect is of the same size as a regular compatibility effect with horizontal stimuli, the change was complete. If it is smaller, it is only partial.

The observed compatibility by Böffel and Müsseler (2019b) did not follow the orthogonal SRC effect often observed in these paradigms [i.e., an advantage of top-right/bottom-left SR mappings over the reverse, see e.g., Lippa & Adam, 2001; Cho, Proctor, & Yamaguchi, 2008; Iani, Milanese, & Rubichi, 2014; Lippa & Adam, 2001; Nishimura & Yokosawa, 2006], indicating a close to complete mental representation caused by the presence of the avatar. The responses were faster and more accurate when a right response was paired with a stimulus that was presented to the avatar’s right, while a left response was compatible with a stimulus presented to the avatar’s left. This observation is in line with the concept of visual perspective taking, because the stimulus now produces the same compatibility pattern as if it was actually presented on the participants’ right.

While it seems to be a straightforward explanation that the avatar changed based on how the stimuli were coded, it is also possible that the coding of the response changed from “right” to “up” instead. In this case, the avatar’s right hand could for example point toward the upper position on the screen and, as a result, the right response could have gained the feature “up”. Based on the previous research, we cannot ultimately decide between these possibilities. Therefore, we want to address this issue in the following experiments by using the aforementioned special features of numbers as stimuli, while allowing for perspective-based differences in response coding and analyzing which way to represent the response leads to compatibility. If we observe a compatibility effect based on the avatar’s point of view that indicates response coding from its perspective, these compatibility effects are most likely a result of changes in response coding rather than stimulus coding.

## Experiment 1

**Fig. 1:**
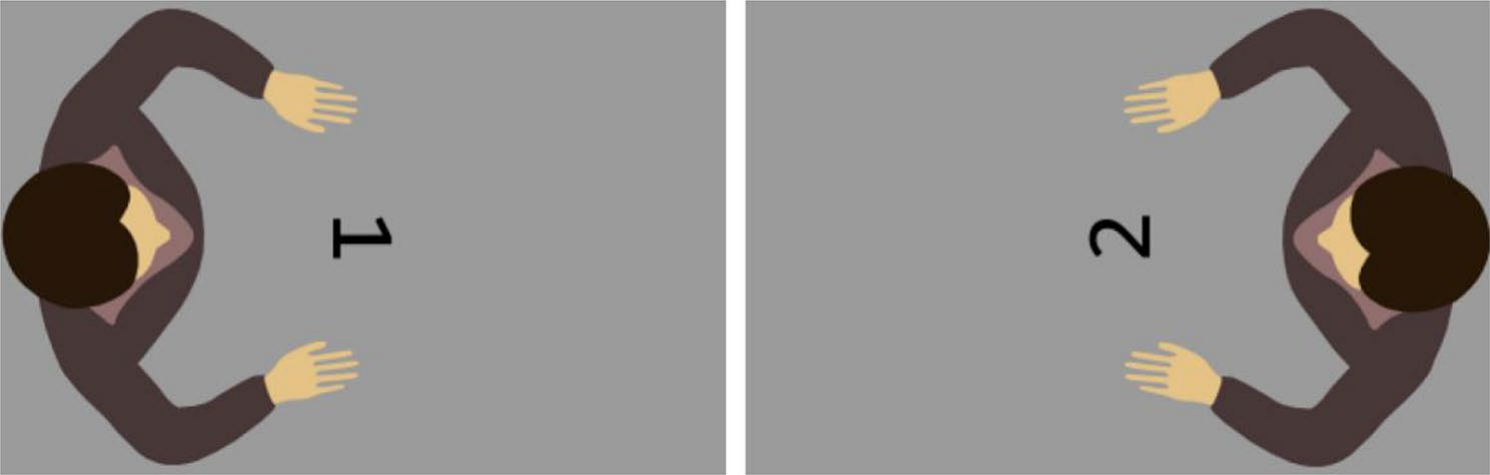
Left: avatar on the left with the target number “1”, an upper response is expected to be compatible from the avatar’s point of view. Right: avatar on the right with target stimulus “2”, a lower response is expected to be compatible from the avatar’s point of view

The first experiment uses numbers presented in front of a top-view avatar to examine the influence of different perspectives on the SNARC effect. In this task, the participants control the avatar so that the avatar always shows a hand movement following the participants’ responses. Both the number and the avatar were rotated 90° to either the left or the right and the avatar’s hand were located above or below a central fixation cross (Fig. 1). The participants performed parity judgments on a sagittally aligned keyboard and the key further away from their body corresponded to the upper position on the screen, while the closer key corresponded to the lower position on the screen.

After each key press, the avatar moved the hand that corresponded to the response position, but not necessarily the response hand. The key that was further away from the participant was mapped onto the top position on the screen, while the closer position was mapped onto the lower position. As a result, the mapping of response key to avatar hand was dependent on the avatar’s rotation. When the avatar is on the right, the closer key (lower response) moved the avatar’s left hand, but when the avatar is on the left, the same key press moved its right hand. There are two possible positions for the participants, and they can have either their left or right index finger perform the lower response. To avoid confusion, the mapping was constant for each participant, but counterbalanced between participants. Because the avatar’s hand movement was defined as the action goal in the instruction, and since action goals are essential for the emergence of stimulus–response compatibility effects (e.g., Hommel, 1993), this setup would lead to response coding on a vertical axis, if it was regarded from the participant’s point of view. Importantly, the avatar itself also provides a frame of reference to code the response that is in conflict with the vertical response coding. For example, when the avatar is on the left, a response that leads to the movement of the lower hand produces a right movement from the avatar’s perspective, while the reverse is true with the avatar being on the right. While the coding of the response can change depending on the avatar’s perspective, the magnitude of the number itself is unaffected. Therefore, changes in compatibility caused by the avatar’s position are likely a result of changes in response coding.

### Hypothesis

We expect that the sagittal SNARC effect is influenced by the avatar’s position as a result of different response coding with different avatar positions. We predict that the avatar’s position leads to a compatibility effect with responses that move the avatar’s left hand being compatible to smaller numbers and responses that move the avatar’s right hand compatible to larger numbers. This means that instead of being coded vertically or sagittally, the responses are expected to be coded from the avatar’s perspective as either right or left. If, however, a compatibility effect arises that is not influenced by the avatar’s position, this would constitute evidence for response coding on a vertical or sagittal axis and indicate that the response code was not changed by the avatar.

## Methods

### Participants

A total of 32 participants (16 female), mainly students from RWTH Aachen University with a mean age of *M* = 25.4 (SD = 5.1), took part in this experiment. All participants reported normal or corrected-to-normal vision and gave informed consent. The sample size was determined based on a rough estimate of effect sizes typically observed in the SNARC paradigm. Dehaene, Bossini, and Giraux (1993) observed a SNARC effect of approximately *η*_p_^2^ = 0.27 and our sample size allows us to detect effects of this size (or larger) with a power of 0.90 (Faul, Erdfelder, Lang, & Buchner, 2007).

### Apparatus and stimuli

We used Matlab and the Psychtoolbox Extension v3.0 (Brainard, 1997; Pelli, 1997) for stimulus presentation and reaction time measurement. The stimuli were presented on a 22″ CRT monitor with a resolution of 1024 × 768 pix at 100 Hz refresh rate. The participants were seated approximately 60 cm in front of the monitor and used a simplified keyboard to perform key presses on a sagittally oriented set of response keys using their index fingers.

The target stimuli were numbers (1–9, excluding 5) in the monospaced Consolas typeface (1.43° × 0.96°) and centrally presented in front of an avatar (8.58° × 7.63°; Fig. 1) which was facing the number. Both the number and the avatar were rotated by 90° either to the right or left from the participants’ point of view, to the 3 or 9 o’clock position, respectively, so that the avatar appeared on the right or left of the screen, facing the screen center. The avatar always moved one of its hands forward after the participant’s key presses by straightening its arm: if the participants pressed the key that was farther away from them, the avatar moved the hand that was closer to the upper edge of the screen, and if the participant pressed the key closer to them (lower key), the avatar moved the hand that was closer to the bottom edge of the screen. In the following, those responses will be labeled as “upper” and “lower” response, respectively. As a result, the avatar moved its right hand after an upper key press in the − 90° conditions, but its left hand in the 90° conditions and so forth.

### Procedure

The participants were instructed to perform a parity judgment and respond to even numbers by producing an upper avatar hand movement and to odd numbers by producing a movement of the avatar’s lower hand. The mapping of even and odd numbers to the response locations and the assignment of the right and left hand to the response keys were counterbalanced between participants. The rotation of the numbers and the avatar changed between the two halves of the experiments with the starting rotation being counterbalanced between participants. The participants performed 19 blocks in each half of the experiment with two presentations of each condition per block for a total of 38 repetitions of each condition and 608 trials. The first block of each half was a practice block and did not enter the analysis. The participants were given the opportunity to take short breaks between blocks.

The avatar remained visible throughout the experiment and each trial started with the presentation of the target number. After the participant responded, the avatar hand movement (a one frame “animation”) was presented as soon as possible on the next frame. False responses as well as responses that were faster than 100 ms (anticipations) or greater than 1500 ms (timeouts) produced an error sound to motivate timely responses. The next trial followed 1500 ms after a response was made and this interval was increased for each error sound by an additional 1500 ms.

### Design

Because every participant only performed one parity mapping, number and response location were collapsed into one factor “sagittal response compatibility”. Based on the assumption of sagittal spatial–numerical associations, key presses far away from the body were categorized as compatible if they were in response to large numbers and as incompatible in response to small numbers. Accordingly, for key presses close to the body, the compatibility was reversed. The result is a 2 × 2 design with the within-subjects factors sagittal response-number compatibility and avatar rotation.

## Results

The first 16 trials of each half were excluded from the data analysis as practice trials. Outliers (5.1% of all trials) were identified and removed from the RT analysis using the Tukey criterion (1.5 × IQR above the third or below the first quartile, respectively). False responses were also excluded from the RT analysis (3.2% of all trials). Mean reaction times and percentage errors (% false responses of all responses) were analyzed separately using 2 × 2 ANOVA with repeated measures on both factors.

### Reaction times and percentage errors

**Fig. 2:**
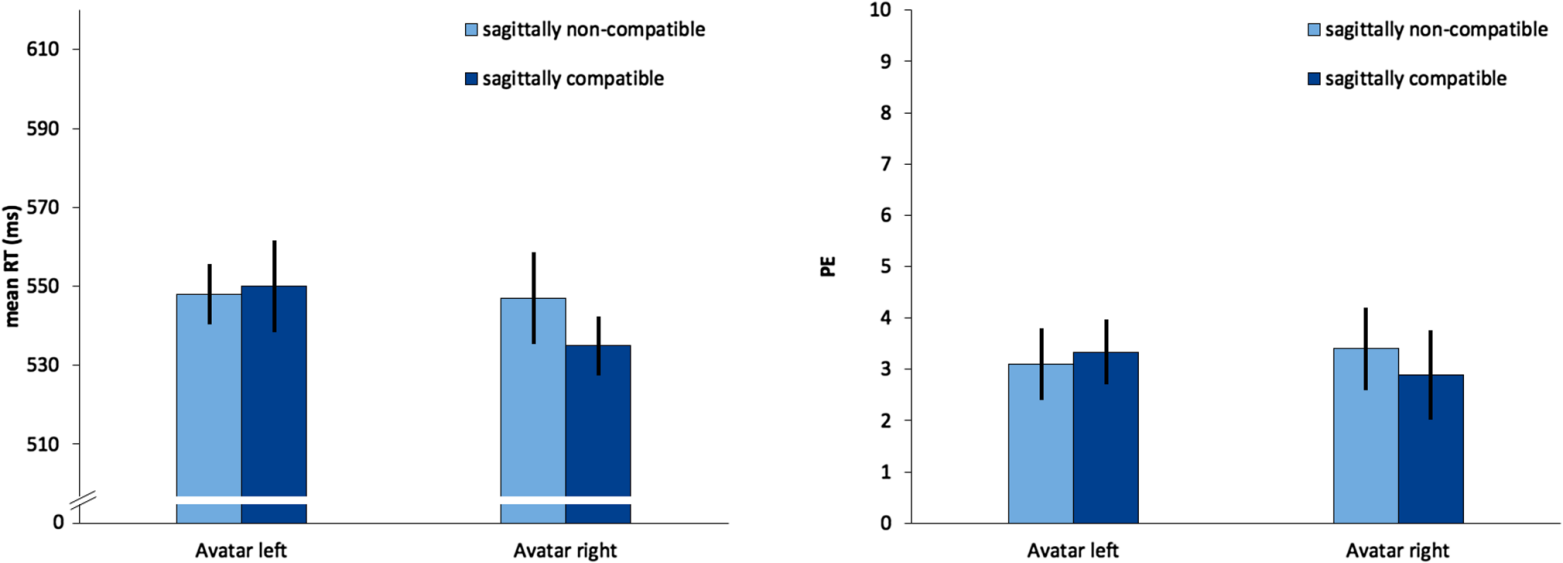
Mean reaction times (RT, left) and percentage errors (PE, right) of Experiment 1 as a function of sagittally SNARC compatibility and avatar rotation. Error bars represent 95% within-subject CIs (Morey, 2008)

We observed a significant interaction of sagittal compatibility and avatar rotation,* F*(1, 31) = 7.25, *p* = 0.011, *η*_*p*_^2^ = 0.190. Sagittally compatible conditions were associated with 11 ms faster reaction times when the avatar was on the right (− 90°). But this effect was changed with an avatar on the left (90°) and sagittally compatible conditions led to 2 ms slower reaction times compared to incompatible conditions. Post hoc *t* tests for repeated measures revealed that the sagittal compatibility effect was significant with an avatar on the right, *t*(31) = − 2.08, *p* = 0.046, while it was non-significant with an avatar on the left *t*(31) = − 0.39, *p* = 0.702. No other significant effects in mean RT were observed. Furthermore, the corresponding analyses of percentage errors revealed no significant effects (Fig. 2).

### Slope analysis

A different approach to analyze the SNARC effect is the use of linear regression analyses for repeated measures data (Lorch & Myers, 1990, Method 3). To do so, for each participant and condition, a linear regression between the number magnitude and the RT differences between both response alternatives (here: far vs. close response) were calculated. These resulting individual regression slopes served as estimates for the individual spatial–numerical associations and can be further statically analyzed (cf. Fias, 1996; Wood, Willmes, Nuerk, & Fischer, 2008).

Since not every participant answered each number with each response location, we grouped the numbers into four ascending magnitude categories: category 1 with the digits 1 and 2, category 2 with 3 and 4, category 3 with 6 and 7 and category 4 with 8 and 9. The individual SNARC slopes for the two avatar rotation conditions (90° rotation, − 90° rotation) were calculated and further analyzed. A paired *t* test revealed that the presence of a SNARC effect depended on the avatar rotation. *t*(31) = − 2.57, *p* = 0.015. When the avatar was on the right, we observed a significant SNARC effect of − 11.4 ms, 95% CI [− 22.5, − 0.2], one-sample *t*(31) = − 2.08, *p* = 0.046. When the avatar was on the left, the effect was 1.8 ms 95% CI [− 11.0, 14.5], one-sample *t*(31) = 0.285, *p* = 0.777.

## Discussion

In this experiment, we used compatibility effects based on numerical–spatial associations to examine whether the presence of an avatar leads to perspective-based changes in response coding. We asked participants to perform a parity judgment of a centrally presented number in the presence of an avatar (Böffel & Müsseler, 2019b). The magnitude of the number in such a task generally causes a compatibility effect resulting in faster responses when large numbers are mapped to right or upper responses and smaller numbers mapped to left or lower responses (Ito & Hatta, 2004; Wood et al., 2008). In contrast to a Simon task with visual stimuli, where the stimulus position can be coded from a different perspective (Böffel & Müsseler, 2019b), the association between numbers and space is based on the magnitude of the number and therefore constant, no matter what perspective is used to view the number. The results are therefore in line with the assumption that the avatar caused changes in the response codes that lead to the responses being perceived as right or left, instead of below or above.

The results showed no overall advantage of sagittally compatible conditions. Instead, compatibility was determined by the avatar’s position. That is, the responses that caused the movement of the avatar’s right hand were compatible with larger numbers and responses that moved its left hand were compatible with small numbers. The results point to the conclusion that a typical, horizontal SNARC effect was observed, but from the avatar’s point of view. Based on the nature of the task, this is most likely a result of changes in response coding. Upper responses were coded as right, with an avatar on the right, and as left, with an avatar on the left. The opposite was true with lower responses. In the conflict of upper/lower versus left/right coding of the responses, the left/right coding appears to be dominant in terms of spatial–numerical associations. This provides further evidence that the preference for an egocentric perspective (Tversky & Hard, 2009) is not universal and can be overcome under the right circumstances. However, looking at the post hoc comparison, between digitally compatible and incompatible conditions depending on avatar perspective, we still see that the effect is larger and only significant with the avatar on the right. This could indicate that the changes in response codes are not complete and that we still have some influence of a sagittal compatibility effect, even though it was not significant. With the avatar on the right, both the compatibility effect from the avatar’s point of view and the sagittal compatibility effect from the participants’ point of view are aligned. The resulting conditions are compatible from both viewpoints and the overall compatibility effect is large and statistically significant. However, with the avatar on the left, both compatibilities are in conflict and conditions that are compatible from the avatar’s point of view are now sagittally incompatible from the person’s own perspective and vice versa. The resulting compatibility effect is smaller and not statistically significant.

The present results are in line with the results of Hommel (1993) in the sense that the action goal is an important factor, but only after it is coded from the avatar’s point of view. In essence, participants had no difficulties in experiencing a response made with their own left hand as right, if the response caused a right hand movement on the screen. One interesting question that follows this observation is whether the action effect has to be present so that the response coding can change, or if the reference frame provided by the avatar alone is sufficient.

## Experiment 2

The previous experiment demonstrated that the compatibility effect based on spatial–numerical associations is dependent on the avatar’s perspective. In Experiment 1, a far key press always produced an upper hand movement, even when this caused a hand-to-hand conflict between avatar and participant. The distal action effect was therefore always visible on screen and the participants showed a pattern of reaction times that indicated that the spatial coding of the responses was dependent on the avatar’s perspective and its hand movements. It therefore remains unclear whether the observed spatial–numerical associations were driven by the sagittal motor features of the responses that are then remapped onto the horizontal axis, or by the visual left/right hand movements. In other words, we do not know if the distal visual action effects on the avatar’s horizontal axes are a necessary condition for a different mapping of numbers and space or if the presentation of an avatar alone would have triggered the perspective taking and affected SNARC effect. The present experiment thus targets the nature of the modulated space–number mapping observed in Experiment 1 and examines the effect of the avatar orientation on the compatibility between numbers and sagittal motor responses in the absence of any visual effects in the avatar.

We expect that the observed influence of the avatar’s perspective is reduced compared to the first experiment.

## Methods

### Participants

A total of 32 (25 female) students of RWTH Aachen University with a mean age of *M* = 21.8 (SD = 3.2) participated in this experiment for course credit. All participants reported normal or corrected to normal vision and gave written informed consent.

### Apparatus, procedure and design

Experiment 2 followed the same method as Experiment 1 with the only exception that no avatar hand movements were displayed after a key press.

## Results

**Fig. 3:**
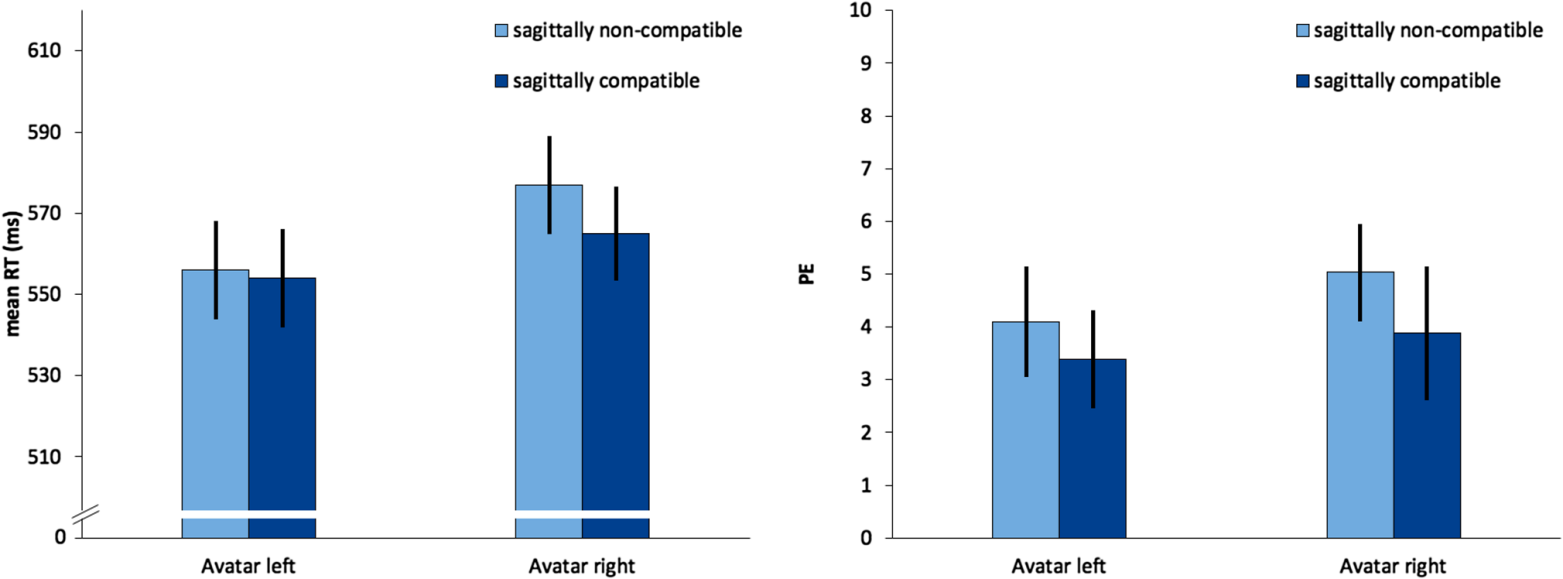
Mean reaction times (RT, left) and percentage errors (PE, right) of Experiment 2 as a function of sagittally SNARC compatibility and avatar rotation. Error bars represent 95% within-subject CIs (Morey, 2008)

Sixteen practice trials were excluded from analysis at the start of each half of the experiment. Reaction time outliers (5.2%) in correct responses were identified using the same criterion as in Experiment 1 and removed from the RT analysis along with false responses (4.1%). Mean reaction times of correct responses and percentage errors were analyzed separately using 2 × 2 within-subjects ANOVA with repeated measures both factors (sagittal compatibility and avatar rotation) (Fig. 3).

### Reaction times and percentage errors

A significant main effect of avatar rotation was observed with 16 ms faster reactions in the avatar left conditions, *F*(1, 31) = 4.43, *p* = 0.044, *η*_*p*_^2^ = 0.125. No other significant effects were observed, but the interaction between sagittal compatibility and avatar rotation approached significance with *F*(1, 31) = 3.07, *p* = 0.089, *η*_*p*_^2^ = 0.090. Here, sagittally compatible conditions were associated with 12 ms faster response times compared to incompatible conditions, when the avatar was on the right. But this difference was only 2 ms with the avatar on the left. Neither of these compatibility effects was significant in post hoc *t* tests with *t*(31) = 1.64, *p* = 0.111 and *t*(31) = 0.40, *p* = 0.694, respectively. No significant effects were observed in the corresponding analyses of percentage errors.

### Slope analysis

The analysis showed that the slopes for both avatar positions were not significantly different from each other with *t*(31) = − 1.39 *p* = 0.174. When the avatar was on the right, we measured a SNARC effect of − 9.1 ms, 95% CI [− 26.4, 8.1], one-sample *t*(31) = 1.08, *p* = 0.289 when the avatar was on the left, the effect was − 1.2 ms 95% CI [− 15.2, 12.8], one-sample *t*(31) = 0.177, *p* = 0.861.

## Discussion

The second experiment examined whether distal action effects are necessary to produce the observed avatar’s influence on response coding in Experiment 1. As a result of the manipulation, the factor sagittal compatibility did no longer significantly interact with the avatar rotation. This independence of the compatibility effect from the avatar’s rotation is also reflected by the slope analysis. While the differences in slopes between both avatar positions follow a similar trend as the results of Experiment 1, the differences are no longer significant. Overall, it seems likely that the presence of distal action effects contributed to the pattern of results in Experiment 1. Numerically speaking, the avatar’s perspective might still have a residual influence on compatibility, so that some changes in response codes are still possible; however, those changes do not reach significance. Therefore, the presence of visible action effects is likely a key factor for spatial response coding.

## General discussion

The present study is among the first to demonstrate that the spatial mapping of numbers can be influenced by the reference frame of an avatar. Previous studies that examined the possibility of shared number lines between participants (Atmaca et al., 2008; Hartmann et al., 2019) attribute the other person’s influence to social processes, e.g., mentalizing. However, it is hard to imagine that the same explanation holds up in our current study, as the situation in our experiment gives the participant complete control over the avatar, turning it into a tool-use scenario rather than a social interaction. Past studies that tried to manipulate the perception of an avatar as either independent or controlled found larger avatar-based compatibility effects in the controlled scenario (Böffel & Müsseler, 2018). This increases the likelihood that the mechanisms behind these avatar-compatibility effects are different compared to the social co-representation of another person’s action. Instead, it seems plausible that the avatar becomes part of a person’s self-representation and its movements therefore part of the person’s own action. This idea is supported by studies that demonstrated body ownership of avatars in similar settings (e.g., Böffel & Müsseler, 2019a).

Experiment 1 demonstrates that effects of number–space compatibility in the sagittal responses emerge only, if the avatar was rotated to the right. This sagittal effect cannot be explained by the anatomical connection between the participants’ hands and the avatar’s hands, because the mapping of hands to response keys was counterbalanced between subjects. Instead, the results point toward a compatibility effect between numbers and response positions, as coded from the avatar’s perspective. Small numbers were overall compatible with responses that were left from the avatar’s point of view and large numbers were compatible to right responses, regardless of what hand the participant moved to perform the response. This shows that not vertical visual effects, such as the movement of the upper or lower arm of the avatar alone, are responsible for the interference with the sagittal SNARC effect. Instead, we demonstrated that the differences of movements on the avatar-dependent horizontal axes are the crucial factor for the modulation of the spatial numerical associations.

Our pattern of effects suggests that two different aspects affected the compatibility effects simultaneously and that the effect is caused by an interplay of the sagittal motor response and the visual horizontal effects relative to the avatar. If the avatar was on the right side, the far and close responses were coupled with congruent horizontal left right movements of the avatar. If, however, the avatar was on the left, the horizontal avatar movements were in conflict with the sagittal response with respect to the numerical associations. Interestingly, under the latter condition, no reaction time differences were observed, suggesting that the two opposite compatibilities neutralized each other. However, we did not observe sufficient evidence for a sagittal SNARC effect in total. Therefore, the participants were mostly able to abandon their own frame of reference when taking the perspective of the avatar. One possible reason for this is that the participants’ attention is directed at the screen which strengthens the influence of the reference frame provided by the avatar and causes them to disregard certain aspects of their own body representation, including the information which hand is used to perform the key press. The avatar’s movements seem to be more important than the movement of the person’s own hand. This observation is common in studies of tool use in which participants often abandon the representation of their own hand in favor of the tool’s end points as long as the correspondence between tool and hand movements is sufficiently high (cf. Müsseler & Skottke, 2011; Rieger, Knoblich, & Prinz, 2005; Sutter, Sülzenbrück, Rieger, & Müsseler, 2013).

One important difference compared to previous studies lies in the nature of the stimulus material. While previous studies focused on using tasks with stimuli that were presented at different locations and therefore contained explicit spatial information, this was not the case with the numbers in the present study. The numbers were centrally presented and did not offer this spatial information on their own, regardless of what perspective was used to view them. Instead, the stimulus feature responsible for the observed compatibility effect—the magnitude of the number—was not influenced by the perspective manipulation and it can therefore be viewed as constant between conditions. In contrast to earlier studies that investigated the impact of avatars on spatial compatibility (e.g., Böffel & Müsseler, 2019b), the results cannot be explained by differences in stimulus coding. Conversely, the avatar offered a reference frame to code the responses and that reference frame was different between both avatar positions. We can therefore conclude that the observed manipulation of the SNARC effect by the avatar is likely a result of differences in response coding. This extends the previous research, which was often unable to separate between perspective-based stimulus and response coding and demonstrates that changes in response coding likely contribute to perspective-based compatibility effects.

Experiment 2 showed that the avatar’s effect on the spatial–numerical associations was reduced or possibly absent when no left/right movements of the avatar were present. This finding confirms the importance of action effects for perspective taking (cf. Böffel & Müsseler, 2018, 2019a, b) and shows moreover that the modulation of the sagittal SNARC effects in Experiment 1 was largely driven by visual left/right hand movements of the avatar. The results of the present study lead to the conclusion that the distal effects are mapped onto a horizontal axis, based on the presence of the avatar alone. The recoding of the action effect is therefore the crucial mechanism that leads to the recoding of the response.

Taken together, the finding of avatar-dependent SNARC effects confirms our assumption that participants take the perspective of the avatar and at least partially use this reference frame for the spatial mapping of numbers to responses. Moreover, it suggests that the required reference frame does not necessarily have to be egocentric, but can be based on another agent. In comparison to experiments with real confederates (e.g., Atmaca et al., 2008), it seems that avatars can cause similar effects. However, different mechanisms are likely at work. Instead of the assumption of a shared cognitive representation by mentalizing the mental number line of another person, it seems to be more parsimonious to assume that the individuals’ number representation—including its spatial association—will be projected onto the avatar if they experience control over the avatar and consider it an extension of their own self.

## Conclusion

Overall, the results of this study show that differences in response coding can cause the compatibility changes observed in the avatar compatibility tasks, especially if they are supported by action effects that can be regarded from the avatar’s perspective. This extends the interpretation of previous experiments that generally attributed perspective-based compatibility effects to differences in stimulus coding. Whether manifest action effects are a necessary condition for this to occur is still somewhat unclear, but it seems plausible that they do at the very least enhance perspective-related compatibility effects.
